# Bone Metastases from Gastric Cancer: What We Know and How to Deal with Them

**DOI:** 10.3390/jcm10081777

**Published:** 2021-04-19

**Authors:** Angelica Petrillo, Emilio Francesco Giunta, Annalisa Pappalardo, Davide Bosso, Laura Attademo, Cinzia Cardalesi, Anna Diana, Antonietta Fabbrocini, Teresa Fabozzi, Pasqualina Giordano, Margaret Ottaviano, Mario Rosanova, Antonia Silvestri, Piera Federico, Bruno Daniele

**Affiliations:** 1 Medical Oncology Unit, Ospedale del Mare, 80147 Naples, Italy; emiliofrancescogiunta@gmail.com (E.F.G.); annalisa.pappalardo88@gmail.com (A.P.); davidebosso84@gmail.com (D.B.); laura.attademo@gmail.com (L.A.); cinzia.cardalesi@gmail.com (C.C.); annadiana88@gmail.com (A.D.); antonietta.fabbrocini@gmail.com (A.F.); fabozzit79@gmail.com (T.F.); giopas@email.it (P.G.); margaretottaviano@gmail.com (M.O.); rosanovamario@hotmail.com (M.R.); antonia.silv@libero.it (A.S.); pierafederico@yahoo.it (P.F.); b.daniele@libero.it (B.D.); 2 Department of Precision Medicine, School of Medicine, University of Study of Campania “L. Vanvitelli”, 80131 Naples, Italy; 3 Department of Clinical Medicine and Surgery, University of Naples “Federico II”, 80131 Naples, Italy; 4 CRCTR Rare Tumors Reference Center of Campania Region, 80131 Naples, Italy

**Keywords:** metastatic gastric cancer, target therapy, bone flare, stage IV, treatment, RANK-L

## Abstract

Gastric cancer (GC) is the third cause of cancer-related death worldwide; the prognosis is poor especially in the case of metastatic disease. Liver, lymph nodes, peritoneum, and lung are the most frequent sites of metastases from GC; however, bone metastases from GC have been reported in the literature. Nevertheless, it is unclear how the metastatic sites may affect the prognosis. In particular, knowledge about the impact of bone metastases on GC patients’ outcome is scant, and this may be related to the rarity of bone lesions and/or their underestimation at the time of diagnosis. In fact, there is still a lack of specific recommendation for their detection at the diagnosis. Then, the majority of the evidences in this field came from retrospective analysis on very heterogeneous study populations. In this context, the aim of this narrative review is to delineate an overview about the evidences existing about bone metastases in GC patients, focusing on their incidence and biology, the prognostic role of bone involvement, and their possible implication in the treatment choice.

## 1. Introduction

Gastric cancer (GC) is the third cause of cancer-related death worldwide [1]. In particular, even today, survival is dismal, and only 5.5% of patients diagnosed with metastatic GC are alive at 5 years [2]. Although over the last decades the research in GC has focused on the role of novel and targeted treatments, chemotherapy based on a doublet with platin and fluorouracil remains the standard of care for the first-line therapy in case of metastatic disease without overexpression of human epithelial growth factor 2 receptor (HER2) [3]. To date, trastuzumab is the unique target agent approved for first-line treatment of HER2-positive metastatic GC in addition to the doublet chemotherapy backbone, due to the fact that all the other targeted agents failed to improve survival outcomes in this setting [4,5]. Recently, the immune checkpoint inhibitors have shown promising results in the treatment of first-line metastatic GC [6,7,8]. However, data are preliminary, and the final results of the trials are awaited in order to clarify their role in the first-line treatment of metastatic GC. Therefore, these agents have not been approved by the regulatory authorities yet, and their use is not a standard of care at the time of writing.

In the last decades, one of the most important conceptual achievements in this field is represented by the “continuum of care”, meaning the possibility to treat patients with multiple subsequent lines of therapy in order to obtain longer survivals. In fact, almost 40% of patients receiving a first-line treatment for metastatic disease maintains a good performance status after progression; they are able to receive a second and even a third line of treatment [9,10]. In the second line, a treatment with paclitaxel and ramucirumab is the standard of care in case of patients with good performance status (PS 0–1 according to the Eastern Cooperative Oncology Group (ECOG) scale); otherwise, ramucirumab as monotherapy is the preferred choice in case of patients with ECOG PS 2 [3]. Then, up to date, triflurifine/tipiracil is the unique agent to have shown a benefit as third line treatment in patients with metastatic GC and good PS, representing the standard choice in this field after the approval of local authorities [3]. Thus, moving from the experience in colorectal cancer patients [11], the definition of the right sequence of treatment from the diagnosis is becoming crucial in GC as well [12].

Over the last decades, a better understanding of molecular patterns of metastatic GC led to validated molecular classifications [13,14], indicating that GC is no longer a single entity, while it includes many subgroups with their own peculiarities and different behavior. However, so far, these molecular classifications have not been applied diffusely in the everyday clinical practice. Additionally, there is still a lack of validated prognostic and predictive biomarkers in GC able to guide the choice of a tailored treatment for each patient [15]. Therefore, the treatment algorithm for metastatic GC is often painted according to the patient’s (age, PS, comorbidities, and nutritional assessment) or tumor’s (tumor burden, symptomatic disease, metastatic sites) characteristics. Thus, a multidisciplinary evaluation of each patient is crucial in the treatment decision process.

In this context, it is unclear how the sites of metastases may affect the prognosis. In fact, if the presence of peritoneal disease or of multiple metastatic sites is considered a well-known worse prognostic factor [16], the knowledge about the role of bone metastases or other visceral sites, such as lung, is scant. This could be related to the rarity of bone involvement in GC, representing the fifth metastatic site after liver, peritoneum, lymph nodes, and—according to the series—lung. Additionally, they are often underestimated at the diagnosis due to the lack of specific recommendation for their detection. Thus, bone metastases have been typically searched for only in case of appearance of new symptoms (e.g., pain), and a consistent fraction of them have been recognized only post-mortem during autopsy in case of non-symptomatic disease [17].

Based on this background, the aim of this narrative review is to describe the evidence existing about bone metastases in GC patients, focusing on their incidence and biology, their prognostic role, and possible implication in the treatment choice.

## 2. The Biological Basis of Bone Involvement in Metastatic Gastric Cancer

From a biological point of view, bones are a unique and favored site for metastases from several types of cancer, and complex mechanisms are responsible for the development of bone metastasis [18,19]. The cells mostly responsible for bone metabolism are osteoblasts, osteoclasts and osteocytes. In general, osteoblasts produce a bone matrix, called osteoid, which consists of proteins, mainly type I collagen (95%), and they synthesize hydroxyapatite crystals, mineralizing the bone matrix; at the end of their synthetic processes, some osteoblasts are “trapped” into their own matrix and become osteocytes [20]. Osteoblasts, which derived from mesenchymal stem cells, are primarily responsible for bone formation; however, they are also involved in bone destruction [21]. In fact, these cells could be stimulated in both physiological and pathological conditions through the excretion of parathormone (PTH) or, in pathological conditions only, through its related peptide- PTHrP- to overproduce the Receptor Activator of Nuclear factor-κB Ligand (RANK-L) [22]. RANK-L—a member of the RANKL/RANK/osteoprotegerin (OPG) pathway and belonging to tumor necrosis factor (TNF) superfamily—exists both as a transmembrane protein and as a soluble form; it interacts with RANK on the surface of osteoclasts, resulting in their activation via transcription factors [23,24]. Preclinical studies have shown that RANK-L is able to promote the migration of breast cancer cells [25] but also of GC cells. Interestingly, in GC it could interact with epidermal growth factor receptor (EGFR) [26]. Osteoblasts are also able to reduce osteoclastogenic activity by expressing OPG, which is a decoy receptor for RANKL that prevents RANK signaling activation [27].

Osteoclasts are multinucleated cells deriving from the monocyte/macrophage lineage, which is responsible for bone resorption by creating sealed zones. In these zones, they can dissolve bone minerals and degrade extracellular matrix (ECM) bone proteins through the secretion of H+, Cl- and enzymes such as cathepsin K and matrix metalloproteinases (MMPs) [28]. During this process, growth factors—namely bone morphogenetic proteins (BMPs) and fibroblast growth factors—are released from the bone matrix; this release causes the attraction of osteoblasts and the start of a new cycle inside the Bone Remodeling Compartment [29,30]. In adulthood, in physiological conditions, bone remodeling reaches equilibrium between formation and resorption. Nevertheless, the quantity of bone loss is not entirely replaced over the years, causing an unrelenting decline in bone mass with aging [31,32].

Several steps are required for bone metastases development: first of all, cells from the primary tumor should reach bones through systemic circulation; then, they enter the bone microenvironment, especially in areas of the skeleton characterized by high vascularization, such as bones with red marrow [33]. The hypothesis of vascular niches in the bone microenvironment, which promotes the skeletal localization of tumor cells through their extravasation and docking, is supported by the discovery of biological and molecular mechanisms, such as cytokines, adhesion molecules, and skeletal endothelial cells properties [34]. Additionally, the dormancy of disseminated tumor cells, followed by their reactivation and proliferation, is a poorly understood process involved in bone metastases development [35].

Although during metastatic dissemination, the circulating tumor cells could localize in bone marrow virtually in all types of solid tumors [36], differences in the incidence of bone metastases across distinct primary tumor sites have suggested the implication of specific and histotype-related mechanisms. In fact, there are several mechanisms—that are not entirely understood—that could lead to a distinct pattern of metastatization, also according to the heterogeneity of primary tumor. In this context, an analysis on 910 samples from 100 patients affected by renal cancer showed that monoclonal tumors tend to provide a more rapid metastatization to multiple sites. Otherwise, heterogeneous tumors had late progression as single metastatic lesions [19]. Nevertheless, bone metastases from gastrointestinal cancers are quite uncommon [33].

Regarding GC, it is still not very clear how the tumor cells colonize the bone microenvironment. Since bone metastases are the results of hematogenous spreading of cancer cells, preferential ways among venous systems, potentially used by GC cells to reach the skeleton, have not been identified [37]. Angiogenesis, which is involved in gastric carcinogenesis and tumor progression, could play a primary role in bone metastasis growth [38]. Among the actors of tumoral angiogenesis, mast cells positive to tryptase (MCPT) have demonstrated to be positively associated with neovascularization in bone metastases from GC, identifying them as a new potential anti-tumor target [39].

PTHrP has been found to be expressed in several epithelial cancers, including gastric adenocarcinoma [40], where it was found to be overexpressed in moderately and poorly differentiated tumors as well as in metastatic sites [41,42]. However, no association with an increased risk of bone metastases has been reported in gastric adenocarcinomas overproducing PTHrP [42].

RANKL expression in resected GC has been associated to high risk of distant metastases and poor prognosis [43]. However, to date no correlation between RANKL expression and bone metastases development risk has been found in those tumors.

Finally, BMP4 has been described to be overexpressed in GC [44]. In an in vivo model of prostate cancer, BMP4 favors tumor growth in bone by tumor-induced osteogenesis [45]. In GC cells, BMP4 could be responsible for the development of metastasis by enhancing epithelial–mesenchymal transition (EMT) [46]. However, despite of these evidences, the role of BMP4 role in bone metastases from GC should be further elucidated.

## 3. Clinical Overview on Bone Metastases from Gastric Cancer

The skeleton has been considered a typical metastatic site in some kind of tumors, such as breast or prostate cancer [47,48]. However, bone involvement is considered unusual in GC. In fact, the little evidence that exists in the literature has shown that bone metastases occur in metastatic GC patients in a range between 0.9% and 13.4%, according to the study population [17,49,50,51,52,53,54,55,56,57,58,59,60,61,62]. However, it is important to note that these data mostly come from retrospective analysis and case series. Therefore, no prospective evaluation regarding the characteristics and distribution of bone metastasis in GC exists to date.

In general, the most important issues to consider in the knowledge of bone metastases in GC patients are the time of onset (at diagnosis—synchronous, or at the time of progression—metachronous), the type of metastases (osteolytic, osteoblastic, or mixed), their distribution (axial versus appendicular skeleton), the prognostic implications of metastatic bone involvement and correlation with other patients’ and tumor’s clinicopathological characteristics.

### 3.1. Incidence, Onset, Type and Distribution

Moving from the first historical report by Yoshikawa et al. in 1983 [17], showing 33 GC patients with bone metastases, the research has focused on this topic especially over the last decade. Table 1 summarizes the most significant data reported in the literature regarding the descriptive evaluation of bone metastases in GC, including time of onset, type, and distribution. Of note, the articles that were not available in English were not considered in this review.

Among the available studies, a retrospective Italian analysis, which collected the data from 2000 metastatic GC patients treated at 22 centers over 12 years (from 1998 to 2011), is one of the largest multicenter experiences in this context [55]. The trial showed a bone involvement in 10% of metastatic GC patients (208 patients); of them, 28% were diagnosed with bone metastasis (synchronous disease), whereas 62% had a bone progression of disease (metachronous disease). The majority of patients were young (<61 years old: 52.9%), male (66%), showing an ECOG PS 1 (43.9%); the most frequent disease characteristics were the following: intestinal subtype according to Lauren classification (38.9%) [63], grade 3 (81.3%), N2 involvement according to TNM stage (41.5%), and concurrent visceral metastasis (86.3%). It is important to underline that 27% of patients had an ECOG PS 2–3 at the diagnosis of bone involvement; additionally, 33% of patients had a diffuse tumor subtype according to Lauren classification [63]. Regarding the type and distribution of the lesions, the majority of patients included in the study population had multiple bone metastasis, especially in the long bones (52%); those lesions were mostly osteolytic (52% versus 25% mixed lesions and 23% osteoblastic ones).

A subsequent Chinese retrospective analysis evaluated the clinicopathological characteristics of bone metastases in 66 patients with GC treated at one institution over a period of 10 years (2008–2018), representing 11.3% of the entire metastatic GC patients population evaluated in the study (884 patients) [56]. Among them, 45.5% and 54.5% had synchronous and metachronous bone metastases, respectively. The majority of those patients were young male (68.2%) with ECOG PS 0–1 (68.2%), multiple metastatic sites involved (bone and visceral: 84.9%), and multiple bone metastases (84.8%) with prevalent axial distribution (spine: 78.5%; pelvic bones: 68.2%; ribs: 47.0%; lower extremity: 34.8%; sternum: 33.3%; scapula: 31.8%; upper extremity: 21.2%; skull: 19.7%). However, this analysis did not report the type of metastasis (osteolytic, osteoblastic, or mixed). Finally, the majority of tumors was poor differentiated/mucinous/signet ring cells (71.2%) and were located in the antrum (30.3%).

Recently, another Chinese retrospective analysis showed the data from 42,966 GC collected in the American population by Surveillance, Epidemiology, and End Results (SEER) database over a period of six years (2010–2016) [57]. Of them, 1798 patients had bone involvement (4.18%). In addition, in this case bone metastases were more commonly reported in young patients (7.3% in 20–39 years old range), with an inverse correlation with age (5.45% in 40–59 years older, 4.09% in 60–79 years older, and 2.39% in >80 years older subgroup). Additionally, they were more common in men and in tumors showing the following characteristics: grade 3 (odd ratio (OR): 3.93, *p* < 0.001), located to the stomach (antrum versus proximal location: OR = 0.81; *p* = 0.02), diffuse according to Lauren classification [63] (OR: 1.46, *p* < 0.001), with signet ring cells or with nodal involvement (OR: 2.09, *p* < 0.001). These data regarding tumor and patients’ characteristics were consistent with those showed by Qiu MZ et al. in another retrospective analysis from the SEER database including GC treated between 2010 and 2014 [58]. Of note, Liang C et al. [56] reported that the patients who underwent to surgical resection of primary tumors had a lower incidence of bone metastases if compared to the patients who did not receive a surgical approach (0.36% versus 7.6%). Finally, also in this case, patients with bone metastases had concurrent extra-bone metastatic sites and multiple bone sites involved. However, this analysis evaluated neither the type of the lesions and their distribution nor their onset time.

Finally, few anecdotal cases of GC patients with bone metastasis located to the bones of the hand were reported [64,65,66,67,68].

**Figure 1:**
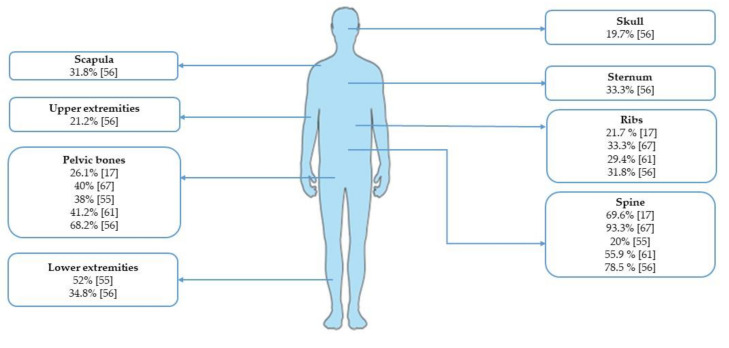
Incidence of bone metastases from gastric cancer according to skeletal sites.

For a summary of the incidence of bone involvement according to different skeletal sites, see Figure 1.

Additionally, there are few reports in the literature regarding the appearance of bone lesions related to early GC [69,70]. When the primary tumor is an early lesion, the presence of metastases to the bone is often underdiagnosed, because bone involvement is investigated only in case of symptoms (e.g., pain). Park et al. focused on the incidence and risk factors of bone recurrence in 1683 GC patients who received a curative resection between 1989 and 2008 [71]. Therefore, this retrospective study analyzed only metachronous bone disease. The incidence of bone involvement was 1.8% in the entire study population, with a higher rate in case of advanced primary tumor at diagnosis (0.4% in case of early GC versus 3.4% in the more advanced stages). The median time from the surgery to the detection of bone metastases was 28 months (range: 4–111 months) and the majority of patients had multiple metastases located to the axial skeleton (spine: 93.3%, pelvic bone: 40% and ribs: 36.6%).

Lastly, even if the majority of the GC recurrence occur into the first two years after curative resection, anecdotal cases of late relapses have been reported in the literature [72,73]. In particular, Iovino et al. reported the case of a 49-year-old patient who showed a tumor relapse from GC after 11 years from the curative surgical resection [73]. The patient had severe neurological signs from a diffuse vertebral involvement and marrow infiltration without detection of any other metastatic lesions or primary tumor; the biopsy of the soft tissue near the lumbar vertebra confirmed the metastatic nature of the lesion, which spread from GC. The authors explained the long latency with the tumor dormancy theory, according to that few tumor cells could be present in the organs in a state of cell-cycle arrest; thus, there is a balance between tumor cells growth and apoptosis that could persist over many years [34]. Even if the entire process is still poorly understood, the tumor cells could reactivate after the variable time, leading to a tumor relapse.

### 3.2. Prognostic Implications of Bone Metastasis in GC Patients

Unfortunately, unlike in other types of tumors, such as renal cancer [74,75], up to date, no prospective dedicated trials regarding GC patients with bone metastases exist in the literature. Additionally, no data about the outcomes of these patients were reported in the landmark phase II and III trials in the metastatic GC setting (see above). Therefore, the most representative data regarding the prognosis of patients with GC and bone metastasis came from the same retrospective analysis already cited in the previous Section 3.1 [17,49,50,51,52,53,54,55,56,57,58,59,60,61,62] (Table 1).

More in detail, Silvestris et al. reported a median overall survival (OS) of 14 months in the entire study population (95% confidence interval (CI): 12–15.9 months), with a median time of 8 months for the first diagnosis of bone involvement (95% CI: 6.1–9.8 months) and a median OS of 6 months from that diagnosis (95% CI: 5–6.9 months) [55]. Of note, they included in the analysis only GC patients with bone metastases who died, whereas they did not evaluate alive patients with documented metastases to the bone. Additionally, they showed that the appearance of skeletal-related events (SREs) impacts on the prognosis (for additional details regarding SRE see the next Section 4.2.2). This analysis showed that a D2 lymph node dissection was an independent prognostic factor of both shorter time to the diagnosis of bone involvement in GC patients (hazard ratio (HR): 2.7, *p*: 0.013) and worse survival in case of bone metastases recognition (HR: 2.285, *p*: 0.008). Finally, there was no difference in median OS in patients diagnosed with synchronous or metachronous bone disease (5 months in each subgroup). This may be related to the worse prognosis linked to the mere diagnosis of bone metastatic involvement. Finally, the authors evaluated only the use of zoledronic acid, whereas there is no mention of the type of chemotherapy used or its impact on survival.

Wen et al. [56] showed a median OS of 6.5 months in the entire population (95% CI: 4.2–8.7 months), with 69.7%, 41.5%, and 13.3% patients alive at 6, 12, and 24 months, respectively. Unlike the previous Italian analysis [55], the author showed a worse OS in case of synchronous disease if compared to metachronous one (4.1 versus 11.8 months, *p* = 0.032). Additionally, they reported a better survival in patients who had received an active chemotherapy (*p*: 0.004) as well as in patients who showed a good PS ECOG (8 months versus 3.6 months in the ECOG 0–1 and ECOG 2 subgroup, respectively; *p* = 0.001). However, the authors did not show the type of active treatment used and the study population was deeply heterogeneous, which was mainly due to the retrospective nature of the study design and the rarity of bone involvement in metastatic GC.

The risk of developing bone metastases was higher in patients affected by metastatic GC with lung involvement (20.24% versus 4.06%, *p* < 0.001) as reported by Qiu et al. [58]. In this analysis, they showed a median OS of 4 months in case of bone metastases, with a case-specific survival rate of 1.27% (versus 29.86% in patients without bone involvement). Therefore, they concluded that even if it is hard to routinely assess a metastatic disease to the bone or the brain from GC, due to the rarity of their involvement and the risk of overlook, it could be feasible to evaluate those sites in patients who have risk factors (e.g., lung or liver metastasis).

The recent analysis by Liang et al. underlined the impact of surgery of the primary tumor site and of the chemotherapy on the outcome of GC patients with bone involvement [57]. In fact, they reported a median OS of 3 months in the entire study population, improved to 9 months in patients who received surgery (versus 3 months in case of no resection, HR: 0.54, 95% CI: 0.40–0.72; *p* < 0.001) and to 7 months in patients who received chemotherapy (versus one month, *p* < 0.001). Of note, this analysis did not show any benefit by adding radiotherapy to the treatment (*p* > 0.05). However, also in this case, the authors did not describe the type of surgery or chemotherapy used, due to the nature of the analysis (retrospective design on data collected in the SEER database). Additionally, they reported the following limitations of the analysis: the underestimation of bone involvement in GC, since they recorded only symptomatic diagnosed cases, the lack of data regarding peritoneal metastasis, and the inaccurate evaluation of death-related causes.

In conclusion, the presence of bone metastases seems to be related to worse prognosis in metastatic GC patients. However, due to the retrospective design of the analysis, the heterogeneity of the study populations, and the rarity of bone involvement, further investigations are needed in order to evaluate the prognostic role of bone metastases and to confirm these findings.

## 4. Clinical Management of Metastatic Gastric Cancer Patients with Bone Involvement

### 4.1. Radiological Assessment

Unlike others cancer types with higher incidence of bone metastases, the European Society of Medical Oncology (ESMO) [3] and National Comprehensive Cancer Network (NCCN) guidelines [76] do not recommend skeletal screening with bone scintigraphy in patients with GC at the time of diagnosis or during treatment. Therefore, the incidence of asymptomatic bone lesions is likely underestimated, and approximately 14% of GC patients are diagnosed with bone metastasis only during autopsy [52].

Chest-abdomen-pelvis computed tomography (CT) scan is the most commonly used imaging technique in the initial diagnostic workup; however, in the follow-up setting, it is recommended only in case of symptoms suspicious for bone recurrence, such as pain [77].

The elevated serum levels of alkaline phosphatase (ALP), which is known to be the most predictive biological marker for the presence of bone metastases in GC, together with the presence of elevated tumor markers, such as carcinoembryonic antigen (CEA) and CA19-9, suggest the need for skeletal screening [78].

In case of recurrence of disease, it has been demonstrated that more aggressive tumor phenotypes, including the presence of lymph node metastasis, are associated with higher risk of bone recurrence. In particular, Park et al. showed that the N2/N3 stage has a risk of bone recurrence significantly higher than N0/N1 (HR: 1.44, 95% CI: 1.217–1.694) [64]. Additionally, Liang et al. have recently described some clinical and histological features than could help the oncologists to identify the “patients with high risk of bone metastases”: Gastro Esophageal Junction (GEJ) cancer, younger age, white race, poor differentiated tumor grade, higher lymph nodes stage, and diffuse histology according to Lauren classification [57,63]. They might suggest the use of radiological bone assessment in this population as standard at baseline.

^18^Fluorodeoxiglucose Positron Emission Tomography (^18^FDG-PET) scan can be useful for the evaluation of lymph nodes involvement as well as for distant metastases including bone metastases. Nevertheless, it is well known that it has a lower sensitivity in GC patients with diffuse histological type, due to the lower glucose transporter 1 (GLUT1) expression in these cells [79].

Finally, the role of magnetic resonance imaging is limited to the evaluation of medullary involvement in the presence of neurological symptoms. Additionally, it is very sensitive for the study of vertebral and pelvic metastatic disease [80].

#### A “Tricky” Evaluation of the Response on Bone Metastases during Treatment: Focus on the Bone Flare

Patients with bone metastases from GC require periodic bone evaluation with a CT scan with a bone window in order to assess the response to therapy according to Response evaluation criteria in solid tumors (RECIST) 1.1 criteria [81]. Additionally, a bone scintigraphy or ^18^FDG-PET could be performed. However, unlike the visceral sites, the response evaluation in case of bone metastases might be difficult due to the existence of strict criteria in this setting [81] and the possibility to detect the bone flair.

The bone flare is a radiological phenomenon, which refers to an increased radiotracer uptake (i.e., 99mTc-labeled bisphosphonates used in the bone scintigraphy) in the bones of the patients with metastatic cancer and bone metastases at baseline, despite the clinical and radiological findings of response to treatment in the other metastatic districts of the body. This phenomenon was firstly studied in prostate cancer patients in the mid-1980s [82]. Indeed, learning from the experiences in prostate cancer, a worsening in bone scan during the first months of systemic treatment for bone metastases could be caused by an intense osteoblastic response and not by a progression of disease [83]. Therefore, it is important to note that we can refer to the bone flair only in case of osteoblastic lesions, whereas an increased isotope uptake by osteolytic metastases should always be considered as a progression of disease. However, often it could be difficult to discriminate bone flare from progressive bone metastases, with a concrete risk of wrong response assessment. In prostate cancer, a prospective study on treatment-naïve metastatic patients showed that bone flare can occur in patients with positive baseline bone scan in a high percentage of cases but also in patients with no abnormalities at the scintigraphy performed at the baseline. Interestingly, these patients were confirmed to have skeletal metastases at follow-up [84].

Bone flare phenomenon has been also reported in other kind of tumors [85,86,87,88], including GC. Up to date, only two case reports about bone flare in GC patients have been published. More in detail, Amoroso et al. reported the case of a 43-year-old man who started chemotherapy for advanced disease. Despite of the appearance of new osteoblastic lesions, treatment was continued, and the second radiological evaluation confirmed the presence of stable osteoblastic lesions [89]. The other case is about a 54-year-old male with baseline bone metastases from GC who showed an increased intensity at bone scan after three cycles of chemotherapy, despite an improvement in skeletal pain. Nevertheless, after additional three cycles of chemotherapy, the bone scan revealed a decrease in the intensity of the signal in the same areas [90].

In conclusion, even if rare, bone flare should be taken into account in metastatic GC patients with osteoblastic lesions who show progression of disease only to the skeleton. In these cases, especially if the patients do not show a clinical worsening, the clinicians should be aware about this phenomenon, since it could be misinterpreted as progression of disease, leading to a change in the chemotherapy regimen. Therefore, a multidisciplinary framework in distinguishing the two conditions is critical also in GC patients.

### 4.2. How to Treat Metastatic Gastric Cancer Patients with Bone Metastases

The treatment of GC patients with metastases to the skeleton can be distinguished into two areas: treatment of metastatic GC disease per se and bone-related treatments.

#### 4.2.1. Systemic Treatments

According to international guidelines [3,76], palliative chemotherapy—with or without targeted agents—is the standard of care in the treatment of metastatic GC. In fact, surgery is not an option in case of metastatic disease, especially to the bone. A detailed description of the currently recommended treatments for metastatic GC disease and/or future perspectives is not in the aim of this review; however, you can refer to dedicated literature and guidelines [3,4,5,12,76] for detailed coverage.

To date, there is a lack of specific data regarding bone involvement in the landmark phase II and III trials in GC. In fact, among ≈50 clinical trials investigating the role of chemotherapy, target therapies, and immunotherapies in advanced GC (first-, second-, ≥third lines) over the last two decades, only seven trials showed specific data for GC patients with skeletal metastases (Table 2).

In particular, among chemotherapy trials, in 2008, Al-Batran S et al. compared the efficacy of FLO (fluorouracil, leucovorin, and oxaliplatin) and FLP (fluorouracil, leucovorin, and cisplatin) regimen as first-line treatment for 220 metastatic GC patients, reporting the data of 6.8% of patients with bone involvement [91]. However, the trial did not show the subgroup analysis for those patients and did not report the outcomes accordingly. In 2009, the non-inferiority phase III ML17032 trial, which compared CX (cisplatin and capecitabine) versus CF (cisplatin and fluorouracil) as first-line treatment in 316 metastatic GC patients, showed a bone involvement in 6.3% of patients; additionally, the authors evaluated the impact of bone metastases on survival outcome (subgroup analysis), showing a benefit by using the CX regimen in these patients (see Table 2 for additional details) [92]. Finally, the phase III SOS trial reported 3.2% prevalence of bone metastases in 625 Asian patients receiving Cisplatin+S-1 every five weeks versus Cisplatin+S-1 every three weeks as first-line treatment [93]. However, no data regarding the impact of bone involvement on the prognosis were shown in this trial as well.

The Korean trial is the only second-line chemotherapy trial reporting the rate of bone involvement in the study population (6%) [94]. No data about the prognosis are available from this trial.

In the context of target therapies trials, the AVATAR [95] and INTEGRATE [96] are the only studies reporting the rate of skeletal metastases. The phase III AVATAR trial investigated the efficacy of the addition of bevacizumab (anti-vascular endothelial growth factor agent) to chemotherapy (CX regimen) as first-line treatment in 202 Asian patients with metastatic GC [95]. The phase II INTEGRATE trial evaluated the activity of regorafenib versus best supportive care as second or third-line treatment for 147 metastatic GC patients [96]. The trials showed that 3.5% [95] and 10.8% [96] of patients had metastatic bone disease, respectively. However, unfortunately, there are no data regarding the prognostic effect of bone involvement in both trials.

Regarding the trials investigating the role of immunotherapy in metastatic GC, the Asian phase III ATTRACTION-2 trial, which tested the efficacy of nivolumab in highly pretreated patients, is the only one showing data about the rate of bone metastases (2.2%) [97,98]. However, also in this case, no data about the outcomes are available for these patients.

In conclusion, even if retrospective evidence seems to suggest a possible worse prognosis in patients with metastatic GC and bone disease, there are no randomized prospective trials confirming these findings. Additionally, no specific data regarding targeted-bone treatments in metastatic GC are available. Therefore, to date, the treatment of metastatic disease does not change according to the metastatic sites involved [3,76]. In particular, the data regarding the impact on bone involvement as well as the response of bone metastases to chemotherapy are limited, and further evaluations about the molecular mechanisms and landscape of bone invasions are needed in order to design specific trials. A multidisciplinary management of those patients, including bone-related treatments, is mandatory in order to palliate the symptoms and to preserve a good PS.

#### 4.2.2. Skeletal-Related Events and Bone-Related Treatments

In general, the presence of bone metastases causes a reduction of physical functions of the patients and deterioration of their quality of life (QoL), especially in case of axial skeleton involvement with multiple osteolytic lesions. In fact, they are often the cause of consistent bone pain that requires opioid-based therapy [139]. Additionally, the presence of bone metastases results in significant morbidity for the patients, mainly because of the associated SREs, which are defined as “pathologic fractures, the need for radiotherapy or surgical interventions to treat or prevent an impending fracture, spinal cord compressions, and hypercalcemia” [59].

In the largest multicenter retrospective study on bone metastases in GC, 31% of patients with skeletal involvement experienced at least one SRE [55]. In this analysis, radiotherapy treatment of the bone lesions was the most common SRE (47.1% of all events), followed by pathologic fracture (22.4%), surgery to the bone sites (15.3%), spinal cord compression (10.6%), and hypercalcemia (4.7%).

Regarding the prognostic value of SREs, median survival in the whole population was 3 months after the development of the first SRE (versus 5 months in patients without SREs), suggesting that the poor prognosis of these patients is related not only to the presence of bone disease per se but also to the appearance of SREs.

Therefore, the bone-targeted treatments are important as well as the systemic therapies in order to improve the outcomes for metastatic patients, allowing them to control pain and to maintain a good PS and eventually receive multiple lines of treatment for metastatic disease (“continuum of care” concept). In this context, radiotherapy, surgery, and drug therapies represent the main options; there is no difference in those treatments according to the primary tumor site (e.g., prostate, breast, gastric, etc.) [140].

The use of radiotherapy has been shown to be very effective to control pain—especially with a neuropathic component—in patients with bone metastases after failure of or intolerance to opioid-based therapy as well as to prevent impending fractures. Several randomized trials have been conducted to assess the fraction schedule of the palliative radiotherapy; based on these analyses and a systematic review [141], a single dose of 8 Gy seems to be the best option for patients with poor prognosis, especially if they have vertebral involvement.

In selected cases, also, orthopedic surgery and neurosurgery can help to improve the QoL of these patients, to control incoercible pain, to prevent long bones and vertebral impending fractures and spinal cord compression.

The role of “bone-focused” drugs has been defined in the last years. Bisphosphonates and RANK-L inhibitors are the most important agents in this field. Bisphosphonates-based treatments, such as zoledronic acid at the dose of 4 mg every 3 or 4 weeks as intravenous infusion, is able to modify the bone homeostasis, with the inhibition of osteoclast-mediated bone resorption; they are useful to prevent and delay SREs in solid tumors, including GC [142]. Silvestris et al. showed a significant extension of the time to the first SRE and an increase in the median survival by using zoledronic acid after the diagnosis of bone metastases in GC patients [55]. These interesting retrospective data may support the beneficial effects of zoledronic acid in GC patients with bone involvement, especially in case of osteolytic lesions. However, further prospective analyses are needed in order to confirm this assumption. Denosumab is a well-studied agent among RANK-L inhibitors. Recently, a combined analysis of three randomized phase III trials has shown that the receptor activator of NF-κB ligand inhibitor Denosumab—used at the dose of 120 mg as subcutaneous injection every 4 weeks—is superior to zoledronic acid in delaying the time to first SRE appearance and in reducing the risk of SRE itself, also in patients with bone metastases caused by cancers different from breast, prostate, lung, and kidney [143]. The optimal duration of treatment with bone-related drugs is not well-established.

Another important issue in the management of bone metastases is the risk of hypercalcemia. In this case, the patient should receive a medical treatment according to guidelines for hypercalcemia stratified for serum calcium levels [144].

Finally, the “hungry bone” syndrome is worth mentioning. This syndrome typically occurs after curative surgery for hyperparathyroidism [145]. However, it could be diagnosed also in anecdotal cases of osteoblastic bone metastases by the occurrence of severe hypocalcemia, which is mainly related to excessive calcium apposition into the osteoblastic massive lesions. Recently, Sakai et al. reported the case of an 87-year-old man diagnosed with GC and bone involvement due to the appearance of hypocalcemia [146]. The treatment of the hungry bone syndrome is the correction of hypocalcemia.

In conclusion, although there are limited evidences regarding the efficacy of zoledronic acid and denosumab in patients with bone metastases from GC, the use of these drugs could be helpful also in GC in order to control the spread of the disease, to improve the life expectancy and QoL, and to reduce the probability of occurrence of SREs. Since no specific data are available up to date, the choice of the best drug should be based on the cost–benefit ratio and toxicity profile.

## 5. Conclusions and Future Perspectives

GC is a very heterogeneous disease; this quite recent concept has become diffusely accepted today. However, the journey to discover the molecular mechanisms that control GC behavior has just started. Recently, the molecular classifications of GC have depicted a very complex landscape [13,14] and multiple molecules, which could be eventually targeted by specific treatments, have been identified [4,5]. Among those molecules, the investigation regarding the role of antiangiogenic agents and multikinase inhibitors, such as cabozantinib, might be interesting future frontiers in the control of bone metastasis also in GC, as shown in other types of tumors, such as renal cancer [147]. However, at the moment, these classifications are hardly applicable in clinical practice. Additionally, there is a lack of knowledge about the molecular mechanisms that control the process of GC metastatization.

In this context, the bone metastases from GC represent still a challenge for the research in this field. In fact, they are rare and often underdiagnosed due to the lack of specific recommendation for their detection according to international guidelines [3,76]. However, bone involvement should be evaluated not only in patients with bone pain or neurological symptoms but also in metastatic GC patients with risk factors, such as aggressive disease or lung metastases. Additionally, there is a lack of prospective evidences regarding specific treatments for patients with bone metastases as well as data showing the outcomes of patients with skeletal metastases from GC or the response of those lesions to standard therapies. Therefore, since the majority of the data in the literature are retrospective and based on a very heterogeneous populations, further prospective studies are needed in order to define the best treatment for GC with bone metastases. Additionally, a better understanding of the underlying molecular mechanisms, by analyzing tumor cells as well as inflammatory tumor infiltrating cells or bone matrix compounds into the bone lesions specimens, could be useful in order to design specific trials.

## Figures and Tables

**Table 1:** Clinical overview about bone metastases characteristics in gastric cancer patients.

Author and Year *	Study Design, Timeline, Country	N Patients Analyzed	N Patient with Bone Metastasis (%)	Onset	Type	Distribution	Main Patients’ Characteristics	Main Tumor’s Characteristics	Outcomes
Yoshikawa et al., 1983 [17]	Retrospective Monoinstitutional 1970–1979 Japan	1945	23 (1.2%)	NR	NR	Thoracic vertebrae: 69.6%; Lumbar vertebrae: 69.6%; Pelvic bones: 26.1%; Ribs: 21.7%	Young (age <60: 78.3%); male: 56.5%	NR	NR
Park et al., 2011 [59]	Retrospective Monoinstitutional 1998–2008 Korea	8633	203 (2.4%)	Synchronous: 62%; Metachronous: 38%	NR	NR	Median age: 51 years; multiple metastatic sites (bone and visceral): 84.7%; multiple bone metastasis: 88.7%; ECOG PS 0–2: 82%	Poorly differentiated: 72%	mOS: 3.4 months
Park et al., 2013 [64]	Retrospective Monoinstitutional 1989–2008 Korea	1683	30 (1.8%)	Metachronous: 100%	NR	Vertebrae: 93.3%; pelvic: bones: 40%; ribs: 33.3%	young (median age 53.1 years old) male: 63.3%	Undifferentiated: 73% N3: 43.3%	mOS after bone recurrence: 6 months
Silvestri N et al., 2013 [55]	Retrospective Multicenter 1998–2011 Italy	2000	208 (10%)	Synchronous: 28%; Metachronous: 62%	Osteolytic: 52%; mixed: 25%; osteoblastic: 23%	Long bones: 52%; Hip: 38%; Spine: 20%	young (median age 61 years old): 52.9%; male: 66%; ECOG PS 0–1: 43.9%; multiple metastatic sites (bone and visceral): 86.3%; multiple bone metastasis: 68.6%	Intestinal: 38.9%; G3: 81.3%; N2: 41.5%.	mOS: 14 months; mOS from the diagnosis: 6 months; mOS SRE versus no SRE: 3 versus 5 months
Nakamura et al., 2014 [60]	Retrospective Monoinstitutional 2000–2010	1837	31 (1.7%)	Synchronous: 25.8%; Metachronous: 74.2%	NR	NR	Age <65: 51.6%; multiple metastatic sites (bone and visceral): 79.5%; multiple bone metastasis: 79.5%; ECOG PS 0–1: 58.1%	Undifferentiated: 67.8%	mOS: 3.3 months
Mikami et al., 2017 [61]	Retrospective Monoinstitutional 2010–2015	NR	34 (100%)	Synchronous: 29.4%; Metachronous: 70.6%	NR	Thoracic vertebrae: 55.9%; Pelvic bones: 41.2%; Lumbar vertebrae: 38.2%; Ribs: 29.4%	multiple metastatic sites (bone and visceral): 76.5%; multiple bone metastasis: 64.7%	Undifferentiated: 55.9%	mOS: 7.5 months
Qiu et al., 2018 [58]	Retrospective Multicenter 2010–2014	19022	966 (5.1%)	NR	NR	NR	NR	Intestinal: 62%; G3: 60.7%; located to the cardia: 38%	mOS: 4 months; 5 year CSS: 1.27%
Wen L et al., 2019 [56]	Retrospective Monoinstitutional 2008–2018 China	884	66 (11.3%)	Synchronous: 45.5%; Metachronous: 54.5%	NR	Spine: 78.5%; pelvic bones: 68.2%; ribs: 47.0%; lower extremity: 34.8%; sternum:33.3%; scapula: 31.8%; upper extremity: 21.2%; skull: 19.7%	young (median age 53 years old) male: 68.2%; ECOG PS 0–1: 68.2%; multiple metastatic sites (bone and visceral): 84.9%; multiple bone metastasis: 84.8%	G3/mucinous/signet ring cells: 71.2%; located to the antrum: 30.3%	mOS: 6.5 months; mOS metachronous: 11.8 months synchronous: 4.1 months
Liang C et al., 2020 [57]	Retrospective Multicenter 2010–2016	42966	1798 (4.2%)	NR	NR	NR	multiple metastatic sites (bone and visceral): 52.6%	Intestinal: 60.8%; G3: 62.2%; located to the cardia: 38.4%	mOS: 3 months
Imura et al., 2020 [62]	Retrospective Monoinstitutional 2005–2017	NR	60 (100%)	NR	NR	NR	Age >60: 56.7%; multiple metastatic sites (bone and visceral): 61.7%; multiple bone metastasis: 83.3%; ECOG PS 0–2: 70%	NR	mOS: 9 months

* listed by year. Abbreviations: N: number; ECOG PS: performance status according to ECOG scale; G3: grade 3; mOS: median overall survival; SRE: skeletal-related events; NR: not reported.

**Table 2:** Data regarding bone metastases according to the landmark phase II/III trials in metastatic gastric cancer.

Trial and Year of Publication	Phase	Setting	*N* Patients	Type of Metastatic Sites Reported (% of Patients)	N Patients with Bone Metastases (%)	Arms and Eventual Target	Outcomes in Patients with Bone Metastases
Chemotherapy *							
V325 trial, 2006 [99]	III	First line	445	NR	NR	TCF versus CF	NR
Dank M et al., 2008 [100]	III	First line	333	Lung: 8.7%; Liver: 49%; Lymph node: 62.1%; Peritoneum: 24.3%; Pleura: 8.1%; Adrenal: 6.3%	NR	CF versus Folfiri	NR
REAL-2, 2008 [101]	III	First-line	1002	NR	NR	ECF/ECX versus EOF/EOX (non-inferiority)	NR
Al-Batran et al., 2008 [91]	III	First-line	220	Lung: 12.7%; Liver: 50.4%; Lymph node: 47.7%; Peritoneum: 30.4%; Pleura: 8.1%; Other: 28.6%	14 (6.8%)	FLO versus FLP	NR
ML17032, 2009 [92]	III	First line	316	Lung: 7.9%; Liver: 48.4%; Peritoneum: 18.7%; Pleura: 3.1%; Soft tissue: 3.8%; Skin: 0.6%	20 (6.3%)	CX versus CF (non-inferiority)	OS: Bone metastases: HR: 1, no bone: HR ≈0.8 (favor CX) PFS: Bone metastases: HR ≈0.6, no bone: HR ≈0.85 (favor CX)
FLAGS trial, 2010 [102]	III	First line	1053	NR	NR	Cisplatin+S-1 versus CF	NR
GC0301/TOP-002 trial, 2011 [103]	III	First line	326	Liver: 33.7%; Peritoneum: 32.2%	NR	S-1 versus S-1+ irinotecan	NR
FFCD trial, 2014 [104]	III	First line	416	NR	NR	ECX versus Folfiri	NR
Yamada et al., 2015 [105]	III	First line	685	Lung: 10.3%; Liver: 36.9%; Lymph node: 84.2%; Peritoneum: 18.2%	NR	Oxaliplatin+S-1 versus Cisplatin+S-1 (non-inferiority)	NR
SOS trial, 2015 [93]	III	First line	625	Lung: 6.8%; Liver: 35.3%; Lymph node: 67.7%; Peritoneum: 37.3%	**20 (3.2%)**	Cisplatin+S-1 q5 weeks versus q3 weeks	NR
AIO, 2011 [106]	III	Second line	40	Lung: 10%; Liver: 45%; Lymph node: 35%; Peritoneum: 45%; Other: 35%	NR	Irinotecan versus BSC	NR
Korean trial, 2012 [94]	III	Second line	202	Peritoneum: 45%; Lung: 9%;; Liver: 28%; Lymph node: 44%	**12 (6%)**	Docetaxel or irinotecan versus BSC	NR
WJOG4007, 2013 [107]	III	Second line	223	Peritoneum: 25.1%	NR	Weekly paclitaxel versus irinotecan (non-inferiority)	NR
COUGAR-02, 2013 [108]	III	Second line	168	Lung: 26%; Liver: 44% Lymph node: 65%; Local: 31% Other: 34%	NR	Docetaxel versus BSC	NR
ABSOLUTE, 2017 [109]	III	Second line	741	Peritoneum: 35.2%	NR	nab-paclitaxel every 3 weeks versus weekly nab-paclitaxel versus weekly paclitaxel	NR
TAGS, 2018 [110]	III	≥ Third line	507	Peritoneum: 27.6%	NR	Trifluridin/tipiracil versus placebo	NR
Target therapy *							
AVAGAST, 2011 [111]	III	First line	774	NR	NR	CX ± Bevacizumab; VEGF	NR
ToGA, 2013 [112]	III	First line	594	NR	NR	CF/CX ± Trastuzumab; HER-2	NR
EXPAND, 2013 [113]	III	First line	904	Peritoneum: 25%	NR	CX ± Cetuximab; EGFR	NR
REAL-3, 2013 [114]	III	First line	553	NR	NR	EOC ± Panitumumab; EGFR	NR
AVATAR, 2015 [95]	III	First line	202	Liver: 39.1%	7 (3.5%)	CX ± Bevacizumab; VEGF	NR
FAST, 2016 [115]	IIb	First line	161	NR (only abstract available)	NR (only abstract available)	EOX ± Claudiximab; claudin 18.2	NR (only abstract available)
LOGIC, 2016 [116]	III	First line	545	NR	NR	CapeOX ± Lapatinib; HER-2	NR
METGastric, 2017 [117]	III	First line	562	NR	NR	Folfox ± Onartuzumab; MET	NR
RILOMET-1, 2017 [118]	III	First line	609	Liver: 41.7%	NR	ECX ± Rilotumumab; MET	NR
HELOISE, 2017 [119]	IIIb	First line	248	NR	NR	CF/CX+ trastuzumab (two doses as mantainance); HER-2	NR
JACOB, 2018 [120]	III	First line	780	NR	NR	CF/CX+ Trastuzumab ± Pertuzumab; HER-2	NR
RAINFALL, 2019 [121]	III	First line	645	Peritoneum: 37.4%; Liver: 29.3%	NR	CF/CX+ ramucirumab; VEGFR-2	NR
GRANITE-1, 2013 [122]	III	Second line Third line	656	Lung: 19.6%; Liver: 45.5%	NR	Everolimus versus placebo; mTOR	NR
REGARD, 2014 [123]	III	Second line	355	Peritoneum: 30.7%	NR	Ramucirumab versus Placebo; VEGFR-2	NR
RAINBOW, 2014 [124]	III	Second line	665	Peritoneum: 47.3%	NR	Paclitaxel ± Ramucirumab; VEGFR-2	NR
TyTAN, 2014 [125]	III	Second line	261	No visceral: 98.8%	NR	Paclitaxel ± Lapatinib; HER-2	NR
INTEGRATE, 2016 [96]	II	Second line Third line	147	Lung: 20.4%; Liver: 53.7%; Lymph node: 51%; Peritoneum: 32%; Other: 36%	16 (10.8%)	Regorafenib versus placebo; multikinase inhibitor	NR
SHINE, 2017 [126]	II	Second line	71	Liver: 56.3%; Lung: 21.1% Peritoneum: 25.4%; Lymph nodes: 54.9%	NR	Paclitaxel ± AZD4546; FGFR-2	NR
GATSBY, 2017 [127]	II/III	Second line	345	Visceral (lung or liver): 100%	NR	Taxanes ± TDM-1; HER-2	NR
GOLD, 2017 [128]	III	Second line	643	NR	NR	paclitaxel ± olaparib; PARP	NR
ANGEL, 2019 [129]	III	≥ Third line	460	NR	NR	Rivoceranib (apatinib) versus best supportive care; VEGFR-2	NR (abstract only)
DESTINY-Gastric 01, 2020 [130]	II	≥ Third line	187	NR	NR	Trastuzumab deruxtecan versus chemotherapy (paclitaxel or irinotecan); HER-2	NR
Immunotherapy (single agent and combinations) *							
Janjigian et al., 2020 [131]	II	First line	37	NR	NR	CF/CX or Folfox/Xelox + pembrolizumab + trastuzumab; PD-1, HER-2	NR
KEYNOTE-062, 2020 [7]	III	First line	763	NR	NR	CF/CX± pembrolizumab or pembrolizumab; PD-1	NR
CHECKMATE 649, 2020 [6]	III	First line	1581	NR (only abstract available)	NR (only abstract available)	Folfox/Xelox± nivolumab; PD-1	NR (only abstract available)
ATTRACTION-4, 2020 [132]	III	First line	724	NR (only abstract available)	NR (only abstract available)	chemotherapy± nivolumab; PD-1	NR (only abstract available)
KEYNOTE-590, 2020 [133]	III	First line	749	NR (only abstract available)	NR (only abstract available)	CF± pembrolizumab; PD-1	NR (only abstract available)
JAVELIN-100, 2020 [134]	III	First line maintenance	805	NR	NR	Folfox/Xelox versus avelumab; PD-L1	NR
EPOC1706, 2020 [135]	II	First line Second line	29	Lymph node: 90%; Liver: 45% Lung: 10%; Peritoneum: 31%	NR	Lenvatinib + pembrolizumab; TKI, PD-1	NR
KEYNOTE-061, 2018 [136]	III	Second line	592	Peritoneum: 28%	NR	Paclitaxel versus pembrolizumab; PD-1	NR
ATTRACTION-2, 2017 [97]	III	≥Third line	493	Liver: 21.5%; Lung: 4.9% Peritoneum: 21.3%; Lymph nodes: 85.8%; Pleural: 1.2%; Adrenal: 0.2%; Other: 10.7%	11 (2.2%)	Nivolumab versus placebo; PD-1	NR
JAVELIN-300, 2018 [137]	III	≥Third line	371	NR	NR	Chemotherapy (paclitaxel or irinotecan) versus avelumab; PD-L1	NR
KEYNOTE-059, 2019 [138]	II	≥Third line	259	Peritoneum: 1.5%	NR	Pembrolizumab; PD-1	NR

* listed by line of treatment and by year of publication. Abbreviations: N: number; NR: not reported; TCF: taxotere/cisplatin/5-fluorouracil; CF: cisplatin/5-fluorouracil; ECF: epirubicin/cisplatin/5-fluorouracil; ECX: epirubicin/cisplatin/capecitabine; EOF: epirubicin/oxaliplatin//5-fluorouracil; EOX: epirubicin/oxaliplatin/capecitabine; FLO: 5-fluorouracil/leucovorin/oxaliplatin; FLP: 5-fluorouracil/leucovorin/cisplatin; CX: cisplatin/capecitabine; CF: cisplatin/5-fluorouracil; OS: overall survival; PFS: progression free survival; BSC: best supportive care; VEGF: vascular endothelial growth factor; HER2: epithelial growth factor receptor 2; VEGFR-2: vascular endothelial growth factor receptor 2; FGFR-2: fibroblastic growth factor receptor 2; PARP: poli ADP ribose polymerase; PD-1: programmed death 1; PD-L1: programmed death ligand 1; TKI: tyrosine kinase inhibitor.

## Data Availability

Not applicable.
